# Study on the Effect of Citric Acid-Modified Chitosan on the Mechanical Properties, Shrinkage Properties, and Durability of Concrete

**DOI:** 10.3390/ma17092053

**Published:** 2024-04-27

**Authors:** Zhibin Qin, Jiandong Wu, Zhenhao Hei, Liguo Wang, Dongyi Lei, Kai Liu, Ying Li

**Affiliations:** 1 School of Materials Science and Engineering, Southeast University, Nanjing 211189, China; qinzhibinseu@163.com; 2 Shandong Provincial Communications Planning and Design Institute Group Co., Ltd., Jinan 250101, China; jdwu_mail@163.com; 3 Qingdao Haifa Real Estate Co., Ltd., Qingdao 266033, China; zhenhaohei@126.com; 4 School of Civil Engineering, Qingdao University of Technology, Qingdao 266033, China; leidongyi@qut.edu.cn (D.L.); liying@qut.edut.cn (Y.L.); 5 Jiangsu China Construction Ready Mixed Concrete Co., Ltd., Nanjing 210033, China; lk230131@126.com

**Keywords:** citric acid-modified chitosan, fresh mix performance, mechanical properties, shrinkage performance, durability performance

## Abstract

As an environmentally friendly natural polymer, citric acid-modified chitosan (CAMC) can effectively regulate the hydration and exothermic processes of cement-based materials. However, the influence of CAMC on the macroscopic properties of concrete and the optimal dosage are still unclear. This work systematically investigates the effects of CAMC on the mixing performance, mechanical properties, shrinkage performance, and durability of concrete. The results indicated that CAMC has a thickening effect and prolongs the setting time of concrete. CAMC has a negative impact on the early strength of concrete, but it is beneficial for the development of the subsequent strength of concrete. With the increase in CAMC content, the self-shrinkage rate of concrete samples decreased from 86.82 to 14.52 με. However, the CAMC-0.6% sample eventually expanded, with an expansion value of 78.49 με. Moreover, the long-term drying shrinkage rate was decreased from 551.46 to 401.94 με. Furthermore, low-dose CAMC can significantly reduce the diffusion coefficient of chloride ions, improve the impermeability and density of concrete, and thereby enhance the freeze–thaw cycle resistance of concrete.

## 1. Introduction

Mass concrete structures frequently experience a cracking issue brought on by temperature stress. Temperature fractures have a number of detrimental impacts on high volume concrete structures, including alkali aggregate reaction, steel corrosion, concrete carbonation, and even a major impact on the durability of concrete, which can result in early building retirement and significant losses [1,2,3]. Therefore, timely control of the shrinkage amplitude of concrete is of positive significance for controlling cracks.

Chitosan, as a natural biopolymer, has good biocompatibility and biodegradability [4,5]. Its degradation products are non-toxic, highly adsorptive, and non-carcinogenic, and are widely used in the biomedical, wastewater treatment, flocculation, and civil engineering fields [6,7,8,9]. The previous literature [10,11] has shown that chitosan, as a polymer structure added to cement-based materials, has great application prospects in improving the early fracture toughness of cement-based materials, improving the rheological properties of cement-based materials, and regulating the heat release process of cement hydration due to the interaction between its molecular chains and the surface of cement particles.

However, chitosan’s effectiveness as a polymer additive in cement-based structures is limited by its insolubility in alkaline pH conditions. Therefore, many scholars have modified chitosan to improve the properties of cement-based materials. The impact of three distinct modified chitosans on cement mortar’s fresh mixing performance was examined by M. Lasheras-Zubiate [12]. The findings demonstrated that the ionic derivative carboxymethyl chitosan (CMCH) postponed the hydration of cement particles and cut the slump of cement mortar by fifty percent. Nevertheless, the slump was only marginally alleviated by the viscosity promoters with a larger molecular weight, these being hydroxypropyl methyl cellulose (HPMC) and hydroxypropyl citrulline (HPC). Bezerra [13] investigated the effects of chitosan and latex on the mechanical properties and durability of concrete. When 2% chitosan and latex were added, the strength increased and polymer fibers were found on the fracture surface of the composite material. Ustinova’s [14] research results showed that the addition of chitosan increased the strength of cement components. The introduction of modified chitosan is beneficial to reducing the overall pore volume in cement components and increasing their frost resistance and bacterial resistance. It was found in our survey of the literature that the application research of chitosan and its modified products in the construction industry is still quite limited. Additionally, they are mostly used as additives and coatings in cement slurry or slurry mixtures. It is worth noting that most studies have focused on using chitosan-modified products as high-efficiency water-reducing agents to investigate their workability, setting properties, etc., [15,16,17] without conducting systematic research on their fresh properties, mechanical properties, or durability. In order to obtain more accurate results to design and control the impact of chitosan and its modified products on the performance of concrete, it is necessary to choose more effective modified products to study the basic properties of concrete, especially to conduct a comprehensive investigation of the fresh-mix performance, mechanical properties, and durability of concrete.

Based on previous research, citric acid-modified chitosan (CAMC) was prepared through acylation reaction and applied to cement-based materials, effectively regulating the hydration process of the cement and, as a result, the rise in temperature and the temperature cracks inside mass concrete [18,19,20]. Therefore, this study systematically investigated the effects of CAMC on the fresh-mix performance, mechanical properties, shrinkage performance, and durability of concrete, providing data support for the further promotion and application of CMAC in concrete.

## 2. Materials and Methods

### 2.1. Materials

P II 52.5 Portland cement (OPC) (CEM PII 52.5 supplied by Xiaoyetian Co., Ltd., Nanjing, China) and a fly ash (FA) (Shenzhen Daote Technology Co., Ltd., Shenzhen, China), as cementitious material, are adopted in this work. The oxide composition of OPC is obtained through XRF testing analysis and the mineral composition of cement is calculated based on the oxide content Bogue model [21], and its basic physical and mechanical properties and composition are shown in Table 1 and Table 2. The auxiliary cementitious material is 2 μm special grade FA. The fineness modulus of river sand is 2.86, the apparent density is 2650 kg/m^3^, and the bulk density is 1650 kg/m^3^. The basalt aggregate has an apparent density of 2720 kg/m^3^, consisting of continuously graded small stones (5 mm~10 mm) and large stones (10 mm~20 mm). The oxide compositions of basalt aggregate and river sand are shown in Table 3. The CAMC used in this experiment was citric acid-modified chitosan prepared in the laboratory. The modification preparation method and the characteristics of the polymer-modified products can be found in references [18,19,20].

### 2.2. Methods

In order to compare the effects of different amounts of CAMC on the fresh mixing performance, mechanical properties, and durability of concrete, 0%, 0.2%, 0.4%, and 0.6% of the cement mass were added to the concrete, with the mixing proportions shown in Table 4.

#### 2.2.1. Fresh Mixing Performance

The slump and spread of fresh concrete are measured using a concrete slump meter, and the test method is based on the standard GB/T50080-2002 [22]. The setting time of fresh concrete slurry with different types and contents of admixtures is measured using a penetration resistance meter, and the test method is JTGE30-2005.

#### 2.2.2. Mechanical Properties

The quasi-static compressive performance test of concrete is conducted on an electric servo hydraulic testing machine with a range of 3000 kN. The compressive strength and tensile splitting strength tests are conducted using cubic specimens (each 100 × 100 × 100 mm^3^). The compressive strength of concrete is determined at a constant loading rate of 0.8–1.0 MPa/s, and the tensile splitting strength of concrete is determined at a constant loading rate of 0.05–0.08 MPa/s.

#### 2.2.3. Shrinkage Performance

The early autogenous shrinkage test of fresh concrete is conducted using the SBT-AS 200 autogenous shrinkage tester. Two parallel samples are formed for each mixing ratio, and the test samples are placed in a corrugated PE tube with a length of 420 ± 5 mm and a diameter of 50 ± 0.2 mm to test the early autogenous shrinkage of the concrete. Then, the sample is placed directly in a standard curing room for curing and testing (temperature of 23 ± 2 °C and relative humidity of 60 ± 2%). The zero point of autogenous shrinkage is based on the initial setting time of the system, which is determined using the penetration resistance method. The calculation method for early autogenous shrinkage strain is shown in the formula:

(1) $$\varepsilon_{a}(t)=\xi (t)-\xi (0)$$

ε_a_(t) is the self-shrinking strain at time t, ξ(t) is the measured linear strain at time t, ξ(0) is the measured linear strain at time 0.

The dry shrinkage performance of the samples was tested according to the GB/T50080-2002 [22]. Three groups of 100 × 100 × 515 mm^3^-prism specimens were made for each mixing ratio sample, and repeated experiments were conducted. After the specimens were formed, they were sent to a standard curing room, and after 48 h of mold curing, the molds were removed. Then, the sample was placed in a standard curing room for curing. The change in the vertical length of the cylindrical specimens was recorded with a microcaliper, and the long-term drying shrinkage rate was calculated.

(2) $$\varepsilon_{d}\left(t\right)=\frac{x\left(t\right)-x\left(0\right)}{l}$$

where ε_d_(t) is the drying shrinkage strain at time t, *x*(t) is the microcaliper measurement at time t (mm), *x*(0) is the initial reading of the test piece placed in the environmental chamber (mm), and l is the initial vertical length.

#### 2.2.4. Durability Performance

(1) Rapid chloride ion migration coefficient method (RCM)

Perform a concrete RCM test according to GB/T 50082-2019 [23], and place the test sample on a concrete chloride ion electrotransportation coefficient tester after vacuum water retention (Beijing RCM-NTB). After power-on, use a 0.1 molAgNO_3_ color indicator solution to test the chloride ion migration depth. After about 15 min, a white silver nitrate precipitate can be seen at the sample interface. Use a marker to draw the color boundary and divide it into 10 equal parts along the interface. Measure the distance from the boundary to the bottom of the sample and take the average as the penetration depth, accurate to 0.1 mm. Calculate the RCM coefficient of the mortar according to Equation (3).

(3) $$D_{RCM}=\frac{0.0239\left(273+T\right)L}{\left(U-2\right)t}\left(X_{d}-0.0238\sqrt{\frac{\left(273+T\right)LX_{d}}{U-2}}\right)$$

where D is the non-steady state ion migration velocity of soil, in 10^−12^ m^2^/s; T is the average of the initial and end temperatures of the anode solution, in °C; U is the absolute value of the applied voltage in the experiment, in V; L is the thickness of the concrete specimen, in mm; X_d_ is the penetration depth of chloride ions, in mm; t is the energization time, in h.

(2) Concrete Freezing-Thawing Cycle Test

Refer to GB/T 50082-2019 [23] for the freezing–thawing cycle test of concrete. Before the freezing–thawing cycle, the test block was first kept in the standard curing room for 24 days, then immersed in water for 4 days to reach saturation. After removing the sample, the surface moisture was wiped clean with a cloth, and the weight was measured and recorded. Then, the test block was placed in a concrete rapid freezing–thawing test machine for the freezing–thawing cycle test. After the test reached the desired number of cycles, the test block was weighed and ultrasonic testing was performed.

(1) Quality loss

The quality loss rate is calculated according to formula (4):

(4) $$\Delta m=\frac{m_{0}-m_{t}}{m_{0}}\times 100\%$$

where ∆m is the mass loss rate of the test block at erosion age t; *m*_0_ is the initial mass of the test block; *m_t_* is the mass of the test block at erosion age t.

(2) Relative dynamic elastic modulus

The dynamic elastic modulus of concrete is measured using a non-metallic ultrasonic tester. Ultrasonic waves with a frequency of 54 Hz are used to test the dynamic elastic modulus of concrete specimens with different erosion ages, and the relative change in the dynamic elastic modulus is calculated. The calculation method can be obtained by using Equations (5) and (6).

(5) $$E=\frac{\left(1+\nu \right)\left(1-2\nu \right)\rho V^{2}}{1-\nu }$$

(6) $$E_{rd}=\frac{E_{n}}{E_{0}}=\frac{V_{n}^{2}}{V_{0}^{2}}=\frac{t_{0}^{2}}{t_{n}^{2}}$$

where E represents the dynamic elastic modulus, in MPa; *E*_0_ and *E_n_* represent the dynamic elastic modulus before and at the erosion age n, respectively, in MPa; E_rd_ represents the relative dynamic elastic modulus, 1; ν represents the Poisson’s ratio of cement-based materials, 1; ρ represents the density of cement-based materials, in kg/m^3^; V represents the ultrasonic velocity, in m/s; t_0_ represents the ultrasonic time before erosion, in μs; t_n_ represents the ultrasonic time at the erosion age n, in μs.

#### 2.2.5. Mercury Injection Pressure Test (MIP)

The pore structure distribution of the hardened paste was tested and analyzed using the AutoPore IV 9500 mercury porosimeter from the American company Microtek. Prior to testing, the test sample is soaked in anhydrous ethanol for termination of hydration, and dried to constant weight in a 50 °C vacuum drying oven. The pore size analysis range was from approximately 4 nm to approximately 300 μm, and high-purity mercury (99.99%) was used for the test at room temperature.

## 3. Results and Discussion

### 3.1. Working Performance 

**Figure 1:**
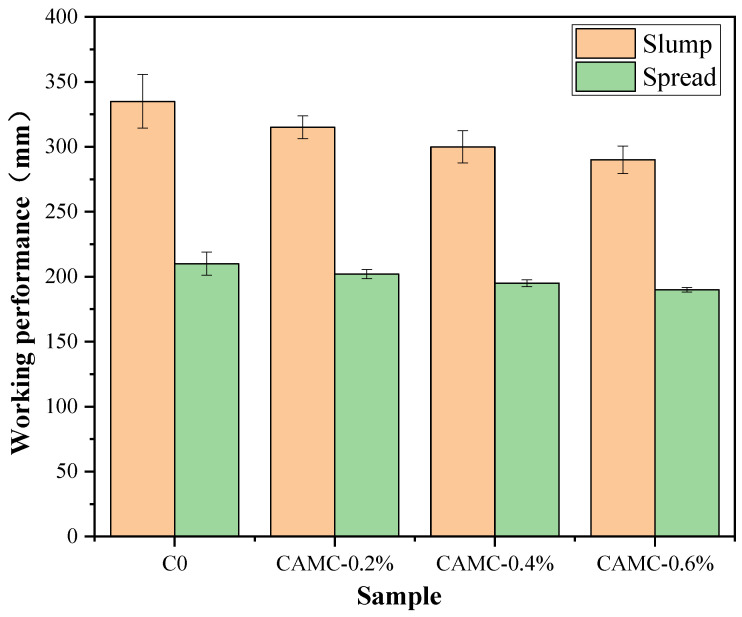
Working performance of premixed concrete.

The change patterns of the slump and the spread of fresh concrete with different CAMC contents are shown in Figure 1. With the change in CAMC content, the slump of the blank group is 335 mm, while the slump of the concrete with 0.2% CAMC added decreases to 315 mm. When CAMC was further added to 0.4% and 0.6%, the slump decreased to 300 mm and 290 mm, respectively. The corresponding spread is 210 mm, 202 mm, 195 mm, and 190 mm. This indicates that, with the increase in CAMC content, the fresh mixing performance of concrete is affected. CAMC has a slight thickening effect. Although CAMC increases the water solubility of chitosan (CTS) and changes its molecular structure, the conclusion obtained from this experiment is the same as that of adding natural chitosan, which also has a thickening effect.

CTS is mainly extracted from chitin and consists of glucosamine and acetyl glucosamine units. When CTS is added, it increases the viscosity of concrete, resulting in a decrease in the fluidity of the mixture [12]. Moreover, its effect is almost independent of the dose. The larger the molecular weight of the CTS, the greater the detected thickening effect, which is caused by the increase in entanglement and crosslinking between chains in calcium-rich systems. The setting time at low doses is mainly affected by the molecular weight of the polymer, while the degree of deacetylation at high doses is the main controlling factor. Due to the interaction between the polymer and the cement particles, CTS in cement mortar also has a retarding effect. However, as a modified derivative of CTS, CAMC has a more significant effect on workability and plays a greater role in thickening.

### 3.2. Setting Time

**Figure 2:**
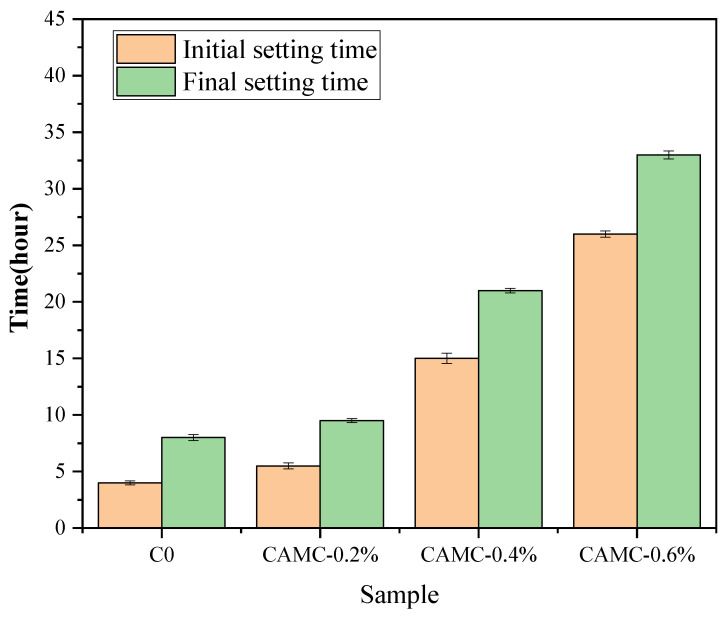
Setting time of fresh concrete.

Figure 2 shows the variation in the setting time of fresh concrete under different CAMC content conditions. It can be observed from Figure 2 that the initial setting time of the blank group concrete is approximately 4 h. After the addition of CAMC, the initial setting times increase to 5.5 h, 15 h, and 26 h, respectively, and the final setting times also increase from 8 h to 9.5 h, 21 h, and 33 h, respectively. The hardening process of cement paste is mainly the formation of a calcium silicate hydrate C-S-H phase. The addition of CAMC leads to the interaction between the CAMC and cement minerals, which delays the diffusion of water to the unhydrated phase, resulting in the delay of the formation of C-S-H and prolonging the setting time. The increase in setting time induced by the presence of CTS and its derivatives has been reported in previous studies [24].

### 3.3. Mechanical Properties

#### 3.3.1. Compressive Strength

**Figure 3:**
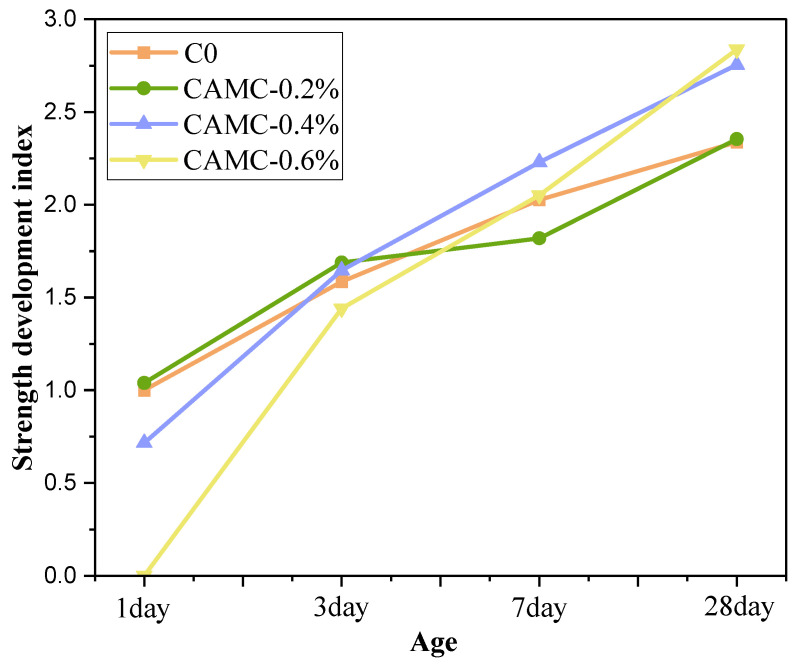
Compressive strength development index of concrete with various CAMC contents at different ages.

The compressive strength of concrete with various CAMC contents at different ages can be found in [18]. Figure 3 shows a strength development index of concrete with different CAMC contents and ages. In the early stage of hardening, CAMC had a negative impact on the initial strength of the concrete. On the first day, the compressive strengths of the C0 and CAMC-0.2% samples reached 21.2 MPa and 22.1 MPa, respectively. As the CAMC content increased to 0.4%, the compressive strength of the CAMC-0.4% sample decreased to 15.2 MPa. After 3 days, the compressive strength values of all samples were above 30 MPa, and the early strength loss caused by the CAMC basically disappeared. It is worth noting that, when the compressive strength was measured at 28 days, the strength of the concrete increased with the increase in CAMC content. The compressive strength of C0 was 49.6 MPa, and the compressive strengths of concrete with added CAMC increased by 0.6%, 17.8%, and 21.4%, respectively. From the strength development index (Figure 3, it can be seen that after 3 days of age, with the increase in CAMC content, the strength of the sample increases more rapidly. This indicated that, although CAMC as an additive added to concrete had a negative impact on early strength, it was beneficial for subsequent strength development.

#### 3.3.2. Splitting Tensile Strength

**Figure 4:**
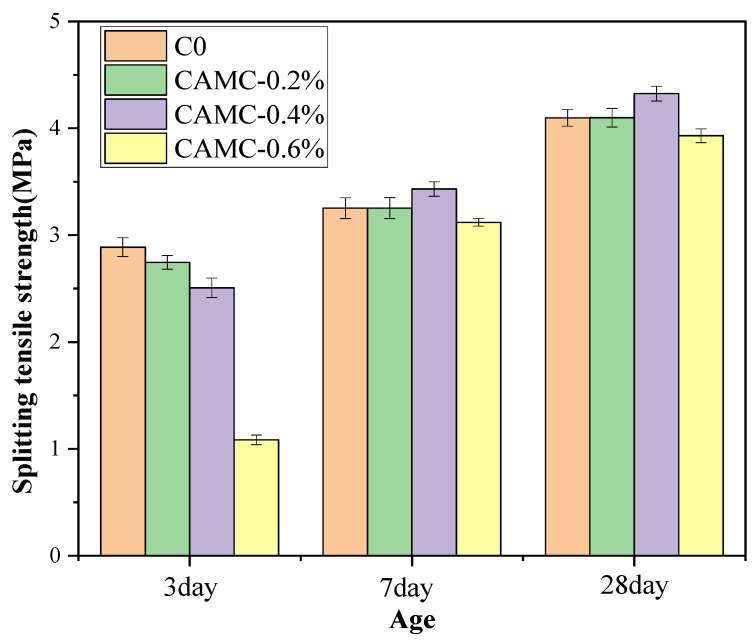
Splitting tensile strength of concrete with different dosages of CAMC.

The splitting tensile strength of concrete under different CAMC content conditions is shown in Figure 4. The figure mainly shows that the splitting tensile strength decreases with the increase in CAMC content in the early stage of hardening, while after the hardening is stable, the splitting tensile strength increases slightly except for CAMC-0.6%. In the early stage of hydration, the splitting tensile strength decreases significantly. At 3 days of age, the splitting tensile strength of the blank group is 2.88 MPa, and after adding different amounts of CAMC, the splitting tensile strengths decrease to 2.74 MPa, 2.51 MPa, and 1.08 MPa, respectively. When hydration progresses to 7 days of age, the splitting tensile strength of the blank group reaches 3.25 MPa, and after adding CAMC, the splitting tensile strengths increase to 3.25 MPa, 3.43 MPa, and 3.11 MPa, respectively. Among the samples, the splitting tensile strengths of higher CAMC content samples increase faster, with CAMC-0.4% increasing by 36.65% compared to 3 days of age, while CAMC-0.6% increases by 188% compared to 3 days of age. This is mainly because, before 3 days of age, CAMC causes a significant delay in the hydration of cement paste, and as the hydration time increases, the hydration rate accelerates and the strength increases significantly. When the curing age reaches 28 days, the splitting tensile strength still has a significant increase compared to 7 days, with the blank group and CAMC-0.2% reaching 4.09 MPa, while CAMC-0.4% increases to 4.32 MPa, and CAMC-0.6% shows a slight decrease compared to the blank group, with a splitting tensile strength of 3.94 MPa. CTS bonds cement particles together through its viscosity, rather than accelerating the production rate of crystallization products. Concrete slump and expansion experiments also show that CAMC increases the viscosity of cement paste while also improving its mechanical strength to some extent. However, when the CAMC content is too high, it can affect the hydration process of cement, which in turn affects the development of later splitting tensile strength.

### 3.4. Shrinkage

#### 3.4.1. Autogenous Shrinkage

**Figure 5:**
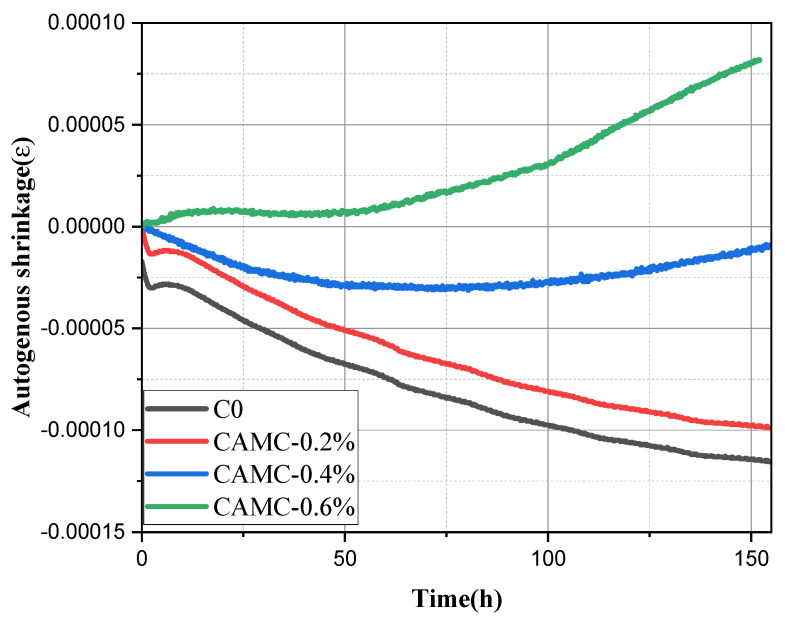
Effect of CAMC on early autogenous shrinkage of cement paste.

The non-contact mortar shrinkage tester was used to detect the autogenous shrinkage development of low-temperature-rise concrete from the initial setting stage to the early hydration stage within 7 days. The test results are shown in Figure 5. From the early autogenous shrinkage variation trend of the four groups of proportions, it can be seen that the first three groups all experienced significant shrinkage in the early stage, while the CAMC-0.6% group experienced a slight expansion phenomenon. Among them, the blank group and the CAMC-0.2% group both experienced a relatively rapid shrinkage at the earliest stage, followed by an expansion behavior. After the expansion reached its peak, the sample experienced a second shrinkage. Based on its early sample shrinkage behavior, the entire change stage of the sample can be divided into three stages: shrinkage, expansion, and then shrinkage [25]. The four characteristic values of ΔP, ΔE, ΔH, and ΔS represent the changes in shrinkage behavior in the three stages, where ΔP is the first-stage shrinkage change value, ΔE is the absolute value of the early expansion phenomenon in the second stage, ΔH is the change value after the expansion reaches its maximum value and then shrinks up to day 7 in the third stage, and ΔS is the final shrinkage change value of the system during the entire early autogenous shrinkage process from the initial setting time to 7 days.

As shown in Table 5, the blank group and CAMC-0.2% group had a shrinkage value of 12.91 and 12.59 με for ΔP, respectively, while the CAMC-0.4% and CAMC-0.6% groups did not exhibit the initial shrinkage phenomenon. When developing into the second stage of early expansion, the blank group and CAMC-0.2% sample group had an expansion value of 15.8 and 18.9 με for ΔE, respectively, with the CAMC-0.2% sample group having a slightly increased expansion value compared to the blank group in the second stage. Similarly, the CAMC-0.4% sample did not exhibit any expansion phenomenon, while the CAMC-0.6% sample had an expansion value of 9.29 με for ΔE. When developing into the third stage of re-shrinkage, it was found that the first three groups of samples had shrinkage values of 87.44, 88.07, and 14.85 με for ΔH, respectively, while the CAMC-0.6% sample continued to expand with an expansion value of 69.2 με. When analyzing the final shrinkage value of the entire system, it was found that the shrinkage of the sample decreased with an increasing CAMC content, with the blank group, CAMC-0.2%, and CAMC-0.4% samples having shrinkage values of 86.82 με, 70.89 με, and 14.52 με, respectively. However, the CAMC-0.6% sample ultimately expanded, with an expansion value of 78.49 με.

**Figure 6:**
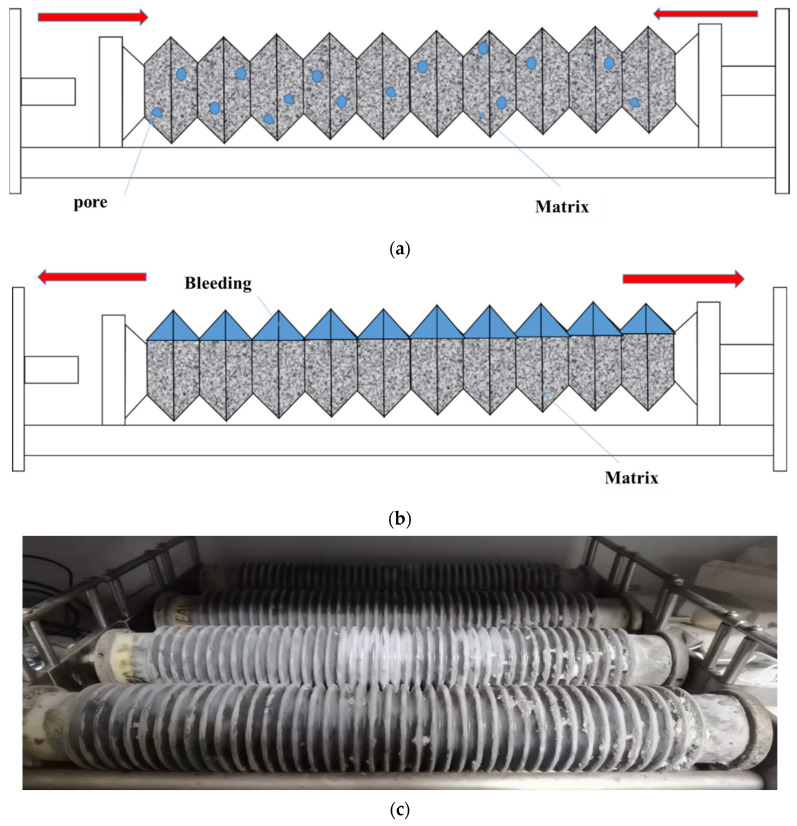
The effect of moisture distribution on the self-shrinking properties of concrete; (**a**) dense slurry, (**b**) bleeding, (**c**) test sample.

There are currently three main theories that can explain the mechanism of autogenous shrinkage: surface tension theory, disjoining pressure theory, and capillary tension theory. The surface tension theory suggests that a decrease in humidity leads to a reduction in the adsorbed water layer between particles within the gelling material, resulting in an increase in surface tension and macroscopic shrinkage of the material [26]. The disjoining pressure theory suggests that the separation pressure between solid particles is a combination of complex forces such as van der Waals forces and layer repulsion. When the humidity decreases, the separation pressure between solid particles decreases and shrinkage occurs [27]. The capillary tension theory suggests that a decrease in humidity leads to the formation of a meniscus at the interface between the gas phase and the liquid phase, resulting in macroscopic shrinkage [28,29,30]. The hydration of cement particles proceeds with the consumption of water and the formation of a porous structure, as shown in Figure 6a. In capillary theory, due to the existence of surface tension, a curved liquid surface forms in unsaturated pores. This meniscus causes capillary stress in the pores, resulting in a decrease in volume [31]. The lower the water–cement ratio, the higher the autogenous shrinkage rate, the finer the pore structure, and the lower the porosity. The consumption of free water gradually causes it to enter small pores from large pores to achieve thermodynamic equilibrium. Due to the significant reduction in pore volume, the consumption of water in low-water–cement-ratio mixtures results in faster changes in pore saturation. This helps to reduce the relative humidity faster in low-water–cement-ratio slurries. In capillary pores with a low relative humidity, the radius of curvature of the meniscus is smaller, so the capillary stress is larger and the autogenous shrinkage Is also larger [32,33,34].

Through the analysis of the above data, it is found that, during the early autogenous shrinkage process, some samples undergo a process of micro-expansion. The expansion source in this stage mainly includes three reasons: first, the expansion pressure caused by the formation of hydration products (CH, AFt), followed by the thermal expansion phenomenon caused by the heat release of hydration [35,36], and then the influence of slurry bleeding and its reabsorption [29,30]. When the sample contains a high content of CAMC, the hydration process is significantly delayed, and there is a significant amount of excess free water in the cement paste, resulting in a “bleeding-like” phenomenon (as shown in Figure 6b,c). Previous ^1^H-NMR research results have also confirmed that CAMC can cause a “bleeding-like” phenomenon [18]. In most cases, the reabsorption of secreted water is the most important reason for this. The free water content and relative humidity inside the high-water–cement-ratio sample are still high, and the capillary stress is relatively small, so the autogenous shrinkage is very small, and the expansion phenomenon occurs [37,38].

#### 3.4.2. Shrinkage Analysis at Different Stages of the Entire Process

**Figure 7:**
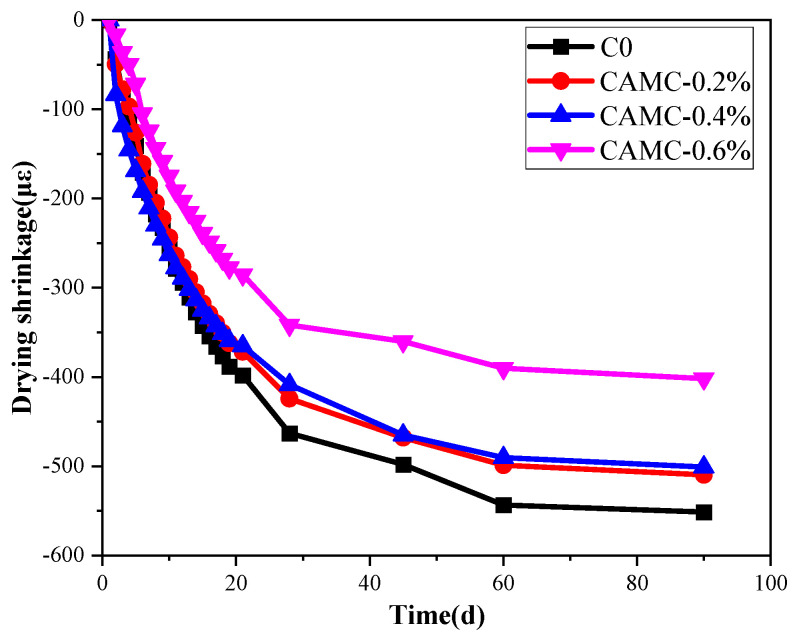
Effect of CAMC on the drying shrinkage of low-temperature-rise concrete.

The drying shrinkage is shown in Figure 7. The long-term drying shrinkage rate of low-temperature-rise concrete decreases with time, and the long-term drying shrinkage gradually decreases with the increase in CAMC content. The drying shrinkage value of the blank group at 90 days is about 551.46 με. After adding 0.2% CAMC, the long-term drying shrinkage value decreases by 42 με. As the CAMC content increases, the long-term drying shrinkage decreases to 500.97 and 401.94 με. The long-term drying shrinkage of the four groups of samples is basically consistent with the change law of autogenous shrinkage described in the previous section.

The overall shrinkage change in low-temperature-rise concrete during the whole process is mainly the sum of the early autogenous shrinkage before demolding and the long-term drying shrinkage after demolding, and was determined in a standard environment. The early autogenous shrinkage and long-term drying shrinkage of low-temperature-rise concrete have been tested and measured in our previous article. Table 6 analyzes the early autogenous shrinkage and long-term drying shrinkage data of the four groups of samples. The specific division method can be found in the literature [25]. A_3_ in Table 6 is the early autogenous shrinkage values of the four groups of samples in Figure 5, A_14_ is the stable autogenous shrinkage values at 14 days, T_14_ is the long-term total shrinkage values of the four groups of low-temperature-rise concrete after demolding at 14 days, and E_14_ is the total shrinkage values of the low-temperature-rise concrete after initial setting. E_14_ can be calculated by the following formula:

E_14_ = A_3_ + T_14_ (7)

D_14_ is the drying shrinkage value of concrete at the age of 14 days, and D_14_ can be calculated by the following formula:

D_14_ = E_14_ − A_14m_ (8)

The analysis of Table 6 reveals that no expansion was observed in any of the low-temperature-rise concrete samples, indicating that the shrinkage caused by drying can balance (or mask) the expansion observed in the early autogenous shrinkage phase. The overall trend is that the total shrinkage value decreases with the increase in CAMC content, where the total shrinkage value of the blank group is 473.37 με, and after the addition of 0.6% CAMC, the total shrinkage value decreases to 370.91 με. The decrease in the total shrinkage of the CAMC-0.6% sample is due to the expansion phenomenon of autogenous shrinkage and the reduction in long-term drying shrinkage. The total shrinkage rate mainly depends on the drying shrinkage rate, as the observed trends are the same in both cases. This phenomenon is due to the larger magnitude of drying shrinkage compared to autogenous shrinkage.

### 3.5. Durability

#### 3.5.1. Chloride Ion Diffusion Coefficient

**Figure 8:**
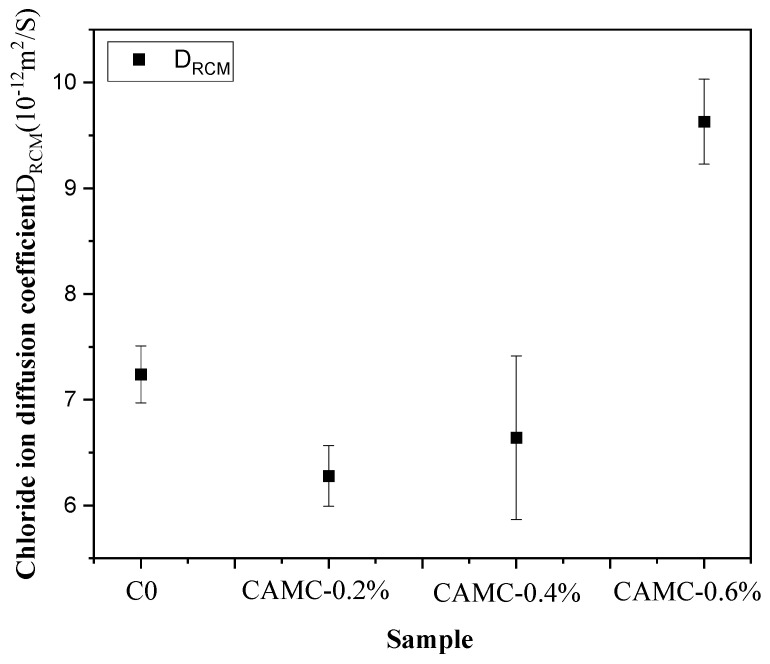
Chloride diffusion coefficients of different samples.

Figure 8 shows the results of the chloride diffusion coefficient of low-temperature-rise concrete samples with different CAMC content. According to Figure 8, the average D_RCM_ of the blank concrete sample is 7.24 × 10^−12^ m^2^/s. With the increase in CAMC content, the chloride diffusion coefficient of low-temperature-rise concrete samples shows a trend of first decreasing and then increasing. The chloride penetration resistance coefficients of low-temperature-rise concrete are in the order of CAMC-0.6% > blank group > CAMC-0.4% > CAMC-0.2%, with average DRCMs of 6.28 ×10^−12^ m^2^/s, 6.64 ×10^−12^ m^2^/s, and 9.63 ×10^−12^ m^2^/s, respectively. Several parameters, such as the water–cement ratio, type of cement and admixtures, curing conditions, the existence of chemical erosion, and characteristics of micro-cracks, can affect chloride penetration. Chloride ions can penetrate into concrete through diffusion caused by concentration gradients and capillary forces, which are related to the volume and size of pores and micro-cracks, as well as their interconnections. Under low-CAMC-content conditions, the chloride diffusion coefficient can be significantly reduced, which plays an important role in improving the durability of low-temperature-rise concrete. By comparing the mechanical properties and shrinkage performance, it can also be found that low-content CAMC can improve the mechanical properties and durability of concrete. Although 0.6% CAMC can still improve the mechanical properties and shrinkage performance of low-temperature-rise concrete in the later stage of hydration, an excessive CAMC content can lead to an increase in the porosity of the concrete structure in the later stage of hydration. The increase in porosity allows salt solutions to pass through the porous regions and cause precipitation in internal pores and voids, resulting in higher permeability of the concrete, leading to an increase in the chloride diffusion coefficient of low-temperature-rise concrete.

#### 3.5.2. Freeze-Thaw Cycle

**Figure 9:**
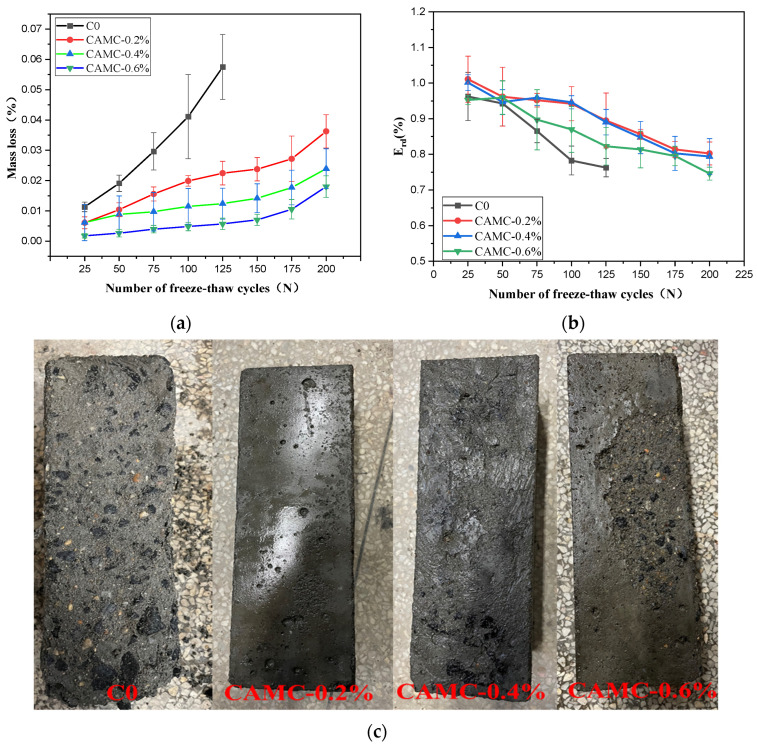
Freeze–thaw cycle test results of samples with different CAMC contents; (**a**) mass loss rate; (**b**) relative dynamic elastic modulus; (**c**) appearance of samples after 150 freeze–thaw cycles.

The quality changes in low-temperature-rise concrete under different freeze–thaw cycles with different amounts of CAMC are shown in Table 7. When the low-temperature-rise concrete is exposed to freeze–thaw conditions, the quality loss in the low-temperature-rise concrete increases with the increase in the number of freeze–thaw cycles. As shown in Figure 9a, the quality loss rate of the low-temperature-rise concrete decreases almost linearly with the number of freeze–thaw cycles. At the same time, the addition of CAMC can reduce the quality loss in the low-temperature-rise concrete. When the number of freeze–thaw cycles reaches 150, the surface of the blank group sample exhibits significant peeling, and the coarse aggregate is exposed significantly, with a quality loss rate exceeding 5% (Figure 9c). When 0.2% and 0.4% of CAMC are added, the appearance of the sample remains basically intact, and no peeling of the sample surface occurs. After 200 freeze–thaw cycles, the quality loss rates are finally 2.39% and 3.62%, respectively. When the amount of CAMC is increased to 0.6%, the surface of the sample begins to exhibit surface erosion, and although the quality loss rate is only 1.79%, it can be observed from the relative dynamic modulus (Figure 9b) that the relative dynamic modulus of the CAMC-0.6% sample after 50 freeze–thaw cycles is lower than that of other samples with different amounts of CAMC. As the amount of CAMC increases, the relative dynamic modulus of the low-temperature-rise concrete shows a trend of first increasing and then decreasing. However, after 125 freeze–thaw cycles, the blank group cannot obtain an ultrasonic wave velocity, indicating that the internal structure of the sample has been damaged.

**Figure 10:**
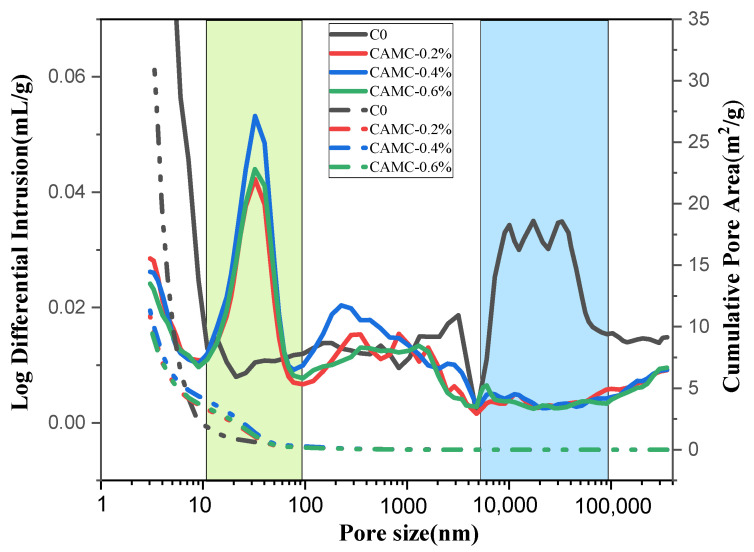
Effect of freeze–thaw cycles on concrete pore structure.

Freeze–thaw cycles can significantly weaken the quality, elastic modulus, and other properties of concrete. The causes of these destructive phenomena can be attributed to water pressure, osmotic pressure, ice crystallization pressure, microscopic ice lenses, and thermal effects that result from the mismatch between ice and solid phases. As the frozen pore solution expands within the pores [39,40] and micro-cracks expand and merge, the connectivity of the pores can be enhanced, further increasing the permeability and accelerating damage. The pore structure determines the degree of freeze–thaw damage, and the pore size distribution of concrete is wide, ranging from 0.5 nm to several centimeters. When air is trapped in the pores, it forms a bubble with a diameter from 10 μm to 1 cm [41]. During hydration, the water in the pores is consumed, and C-S-H gel can be filled into the pores. Therefore, the capillary pore volume decreases while the gel pore volume increases. As shown in Figure 10, the pore structures of the concrete samples after 150 freeze–thaw cycles showed a significant change. The pore diameter of the control group mainly ranged from 10 to 100 μm. With an increase in CAMC, the porosity in concrete decreases, and the pore size distribution mainly ranges from 10 to 100 nm. Compared with the control group, the total porosity decreases from a high of 30% to around 15%, indicating that, after the addition of CAMC, harmful pores (>200 nm) in the concrete matrix gradually transform into less harmful pores (20–50 nm) and harmless pores (<20 nm) [42,43]. The addition of CAMC can increase the impermeability of the concrete matrix and improve the density of the concrete.

## 4. Conclusions

This work systematically studies the influence of CAMC on the fresh concrete properties, macroscopic mechanical properties, shrinkage behavior, and durability of concrete. The main conclusions are as follows:

(1) CAMC enhances the water solubility of CTS, and the interaction between CAMC and cement particles increases the consistency of cement paste. As the amount of CAMC increases, the slump and spreading of concrete decrease. Meanwhile, CAMC prolongs the setting time of concrete. When the content of CAMC is too high, it will affect the hydration process of cement, delay the formation of early C-S-H and hydration products, and have a negative impact on the early compressive strength and splitting tensile strength. However, with the acceleration of the hydration rate in the later stage, it is beneficial to the development of subsequent strength.
(2) The early shrinkage stages of the studied specimens can be divided into three phases: shrinkage, expansion, and re-shrinkage. With the increase in CAMC content, the early shrinkage values of the specimens were 86.82 με, 70.89 με, and 14.52 με, respectively. When the CAMC content was 0.6%, the hydration process was significantly delayed, and a large amount of excess free water in the cement paste led to a “bleeding-like” phenomenon, resulting in expansion behavior with an expansion value of 78.49 με. The total shrinkage value and drying shrinkage value exhibited the same trend. As the CAMC content increased, the shrinkage value decreased. The total shrinkage value of the blank group was 473.37 με, while the total shrinkage value decreased to 370.91 με after adding 0.6% CAMC. The total shrinkage rate was mainly determined by the drying shrinkage rate.
(3) At low CAMC dosages, the chloride ion diffusion coefficient can be significantly reduced, which plays a certain role in improving the durability of low-temperature-rise concrete. The chloride penetration resistance coefficient of low-temperature-rise concrete follows the order: CAMC-0.6% > blank group > CAMC-0.4% > CAMC-0.2%. After adding CAMC, harmful pores in the concrete matrix gradually transform into less harmful and non-harmful pores. However, a high dosage of CAMC can increase the porosity of the concrete structure in the later stages of hydration, leading to an increase in the chloride ion diffusion coefficient of low-temperature-rise concrete. Adding CAMC can reduce the mass loss of low-temperature-rise concrete. With the increase in CAMC content, the relative dynamic modulus of low-temperature-rise concrete exhibits a trend of first increasing and then decreasing.

## Figures and Tables

**Table 1:** Physical properties of OPC.

Density (g/cm^3^)	Specific Surface Area (m^2^/kg)	Water Demand (wt.%)	Initial Setting Time/min	Final Setting Time/min	Flexural Strength/MPa		Compressive Strength/MPa	
				3 d	28 d	3 d	28 d	
3.12	372	30	186	275	5.10	8.15	30.75	54.04

**Table 2:** Chemical and mineral compositions of portland cement and fly ash (wt.%).

Material	CaO	SiO_2_	Al_2_O_3_	Fe_2_O_3_	SO_3_	MgO	K_2_O	Na_2_O	TiO_2_	Loss
OPC	63.62	19.70	4.45	2.93	2.93	1.28	0.68	0.12	0.27	3.92
FA	17.60	65.67	6.84	0.06	-	0.08	0.04	0.035	0.015	9.639
OPC	C_3_S	C_2_S	C_3_A	C_4_AF	Gypsum (CaSO_4_·2H_2_O)	CaCO_3_				
wt.%	63.6	15.1	7.2	6.4	2.9	4.8				

**Table 3:** Oxide composition of basalt aggregate and river sand (wt.%).

Material	SiO_2_	Al_2_O_3_	Fe_2_O_3_	MgO	CaO	Na_2_O	K_2_O	TiO_2_	MnO	P_2_O_5_	Loss
Basalt aggregate	48.77	13.95	13.09	4.86	8.08	2.32	1.40	4.18	0.18	0.38	2.35
River sand	82.47	9.26	2.02	0.557	3.66	0.94	0.83	0.11	-	-	0.16

**Table 4:** Mix proportions of concrete.

Component	C0	CAMC-0.2%	CAMC-0.4%	CAMC-0.6%
OPC (kg/m^3^)	336	336	336	336
FA (kg/m^3^)	95	95	95	95
Natural sand (kg/m^3^)	789	789	789	789
Basalt (kg/m^3^)	1079	1079	1079	1079
Polycarboxylate water reducer (wt.%)	0.2	0.2	0.2	0.2
CAMC (wt.%)	0	0.2	0.4	0.6
Water/binder ratio	0.4	0.4	0.4	0.4

**Table 5:** The characteristic value of early autogenous shrinkage change in cement paste.

Number	ΔP/×10^−6^ m	ΔE/×10^−6^ m	ΔH/×10^−6^ m	ΔS/×10^−6^ m
C0	−12.91	15.8	−87.44	−86.82
CAMC-0.2%	−12.59	18.9	−88.07	−70.89
CAMC-0.4%	-	-	−14.85	−14.52
CAMC-0.6%	-	9.29	69.2	78.49

**Table 6:** Shrinkage value of the whole process of low-temperature-rise concrete mixed with CAMC.

Number	A_3_ (με)	A_14_ (με)	T_14_ (με)	E_14_ (με)	D_14_ (με)
C0	−146.19	−86.82	−327.18	−473.37	−386.55
CAMC-0.2%	−166.78	−70.89	−304.85	−471.63	−400.74
CAMC-0.4%	−170.15	−14.52	−313.59	−483.74	−469.22
CAMC-0.6%	−145.67	78.49	−225.24	−370.91	−449.4

**Table 7:** Mass changes in samples with different CAMC contents.

Sample	Initial Value	Number of Cycles							
		25	50	75	100	125	150	175	200
C0-1	7.98	7.90	7.84	7.80	7.77	7.62	damage	damage	damage
C0-2	7.66	7.56	7.49	7.41	7.33	7.17	damage	damage	damage
C0-3	7.42	7.34	7.29	7.17	7.02	6.95	damage	damage	damage
CAMC-0.2%-1	7.65	7.62	7.58	7.54	7.51	7.50	7.49	7.48	7.41
CAMC-0.2%-2	7.82	7.77	7.75	7.71	7.65	7.61	7.60	7.54	7.49
CAMC-0.2%-3	7.68	7.62	7.58	7.54	7.53	7.52	7.51	7.50	7.41
CAMC-0.4%-1	7.66	7.56	7.54	7.53	7.52	7.52	7.51	7.48	7.42
CAMC-0.4%-2	7.44	7.42	7.39	7.39	7.38	7.37	7.35	7.31	7.28
CAMC-0.4%-3	7.44	7.42	7.41	7.40	7.38	7.37	7.36	7.35	7.30
CAMC-0.6%-1	7.52	7.51	7.50	7.50	7.49	7.49	7.48	7.46	7.39
CAMC-0.6%-2	7.42	7.41	7.41	7.39	7.39	7.38	7.37	7.35	7.31
CAMC-0.6%-3	7.78	7.76	7.75	7.74	7.73	7.72	7.71	7.67	7.61

## Data Availability

Data presented in this study are available on request from the corresponding authors due to restrictions privacy.
